# Impact of Different Types of Sedentary Behaviour on Cognitive Function in Older Adults: A Systematic Review

**DOI:** 10.1002/hpja.70190

**Published:** 2026-04-28

**Authors:** Tawan Ricardo de Jesus Silva, Eduarda Pereira Damião, Vanessa Santos Barbosa, Lara de Matos Alcantara, Rafaela Gomes dos Santos, Jair Sindra Virtuoso Junior

**Affiliations:** ^1^ Graduate Program in Physical Education Federal University of Triângulo Mineiro (UFTM) Uberaba Minas Gerais Brazil; ^2^ Department of Education (DEDC 10) State University of Bahia (UNEB) Teixeira de Freitas Bahia Brazil

**Keywords:** cognition, cognitive ageing, life style, older adults, sedentary behaviour

## Abstract

**Issue Addressed:**

Sedentary behaviour (SB) is a relevant determinant of health in ageing, yet its effects on cognitive function remain inconclusive. The literature often treats SB as a homogeneous exposure, without considering that different sedentary activities may differentially affect cognitive domains. Accordingly, this study sought to identify how distinct types of SB influence cognition in older adults.

**Methods:**

A systematic review was conducted and registered in PROSPERO (CRD42025637160), following PRISMA guidelines. Observational studies published between 2010 and 2025 were included if they involved participants aged 60 years or older and examined the relationship between specific types of SB and cognitive domains. Searches were performed in PubMed/MEDLINE, Web of Science and Scopus. Two independent reviewers carried out study selection, data extraction and quality assessment using Joanna Briggs Institute tools.

**Results:**

The search yielded 1795 records, resulting in the inclusion of 13 studies encompassing 43 902 participants. Findings were heterogeneous: passive SB, particularly, television viewing, was more frequently associated with poorer cognitive performance. In contrast, cognitively stimulating sedentary activities involving complex tasks—such as reading, playing games and computer use—were positively associated with memory, executive function and processing speed. Some studies further suggested that cognitive engagement may mitigate potential negative effects of SB.

**Conclusions:**

The findings indicate that the impact of SB on cognition depends more on the type of activity performed than on total sedentary time, underscoring the importance of context and mental demand.

**So What?:**

Identifying which types of SB are beneficial or harmful may inform health promotion strategies, encouraging older adults to replace passive SB with cognitively stimulating sedentary activities, thereby supporting healthier cognitive ageing.

## 1 Introduction

Human lifestyles undergo continuous changes depending on adopted habits, directly impacting individual health [1], particularly, among older adults, whose routines often change abruptly according to physical, social and economic conditions [2]. When distinguishing the time allocated to daily activities such as work, sleep, eating, leisure and physical activity, it is observed that better adjustments in daily routines positively influence life expectancy [3, 4].

Nevertheless, time spent in sedentary behaviour (SB) is also part of the overall routine [5]. Recent studies have highlighted concerns regarding excessive time spent in this type of behaviour, due to its harmful effects on health and psychosocial determinants [6, 7]. However, when addressing the effects of SB on cognition in older adults, the evidence remains inconclusive. A systematic review with meta analysis demonstrated a positive association between higher levels of SB and increased risk of cognitive decline [8]. On the other hand, Dillon et al. [9] emphasise that the effects of SB depend on the context in which it occurs, suggesting that different types of SB may exert distinct impacts on cognitive domains.

Concurrently, cognitive function comprises multiple subdomains such as language, memory, executive function and attention, each supported by specific neural circuits and susceptible to different influences throughout ageing [10]. Therefore, the stimulation or decline of each subdomain depends on a variety of factors, including the type of activity performed, its complexity and the time devoted to it. Evidence suggests that different patterns of cognitive engagement may either mitigate or accentuate cognitive changes in older adults, reinforcing the dynamic and contextually modulated nature of cognition at this stage of life [11].

Considering that most of the literature treats SB as a single entity, without accounting for the various contexts in which it occurs, this study helps to identify which types of SB have a greater impact on cognitive activity. Thus, the guiding question of this review is: What is the impact of different types of SB on specific cognitive domains in older adults? Accordingly, the objective of this study is to systematically analyse the impact of different contexts of SB, such as watching television, use of electronic devices, reading, games, religious activities, among others, on different cognitive domains in older adults.

## 2 Methods

This systematic review was registered in the International Prospective Register of Systematic Reviews PROSPERO (CRD42025637160) and followed the recommendations of the Preferred Reporting Items for Systematic Reviews and Meta Analyses PRISMA [12].

## 3 Eligibility Criteria

This review included studies whose participants were community‐dwelling older adults aged 60 years or older [13]. Eligible studies were required to report exposure to SB in specific domains, such as television viewing, computer use, reading time, games or similar activities and to provide explicit information on at least one cognitive domain, including memory, language, orientation or other domain‐specific cognitive outcomes.

Studies that included populations with a mean age below 60 years were eligible only when results were stratified by age, allowing extraction of data exclusively for participants aged 60 years or older.

Studies were excluded if they focused on institutionalised or residential care populations, participants with diagnosed dementia or moderate to severe cognitive impairment or clinical samples selected based on specific neurological or psychiatric conditions. In addition, studies were excluded if they did not specify the SB domain assessed, if they reported only total sedentary time or weekend sedentary time or if they evaluated only overall cognitive decline or global cognition defined as a composite score across multiple cognitive domains.

## 4 Search Strategy and Sources

Searches were conducted in the PubMed/MEDLINE, Web of Science and Scopus databases. When applicable, filters were applied to restrict publication dates between 2010 and 2025. The search strategy was based on three key concepts: sedentary behaviour, cognitive function and older adults, using controlled vocabulary and free‐text terms combined with Boolean operators to broaden retrieval (Appendix A).

Only studies published from 2010 onwards were included to ensure conceptual and methodological consistency with the contemporary definition of SB. From this period, studies more frequently distinguished SB from physical inactivity, differentiated specific sedentary activities and used more standardised and validated measurement approaches, improving comparability across findings.

## 5 Study Selection

Observational studies including cohort, case control and/or cross‐sectional designs published over the last 15 years were selected. These studies assessed the impact of different types of SB activities on cognitive function in older adults with a mean age equal to or greater than 60 years. Initially, titles and abstracts were independently screened in pairs, followed by full text assessment for inclusion in the systematic review.

## 6 Data Collection Process and Data Extraction

Two authors TRJS and EPD performed the study selection and identified studies based on titles and abstracts. Duplicate articles and those that did not meet the selection criteria were removed. The remaining articles were reviewed in full text by the same authors to determine eligibility. Any discrepancies and or doubts regarding the manuscripts were resolved by a third reviewer VSB. Both authors TRJS and EPD conducted the search, extracted files to the Rayyan Systematic Review platform and performed selection based on the predefined criteria. The software indicated that the researchers were fully aligned and identified only two conflicts among the studies.

TRJS and EPD independently extracted data from all included articles using a customised extraction table developed by the reviewers in a Microsoft Excel spreadsheet 2024. The following general information was extracted: (1) first author name and year of publication; (2) study design, age range and number of participants; (3) country; (4) study objective in relation to SB and the outcome.

For SB, the following information was extracted: (1) measurement instrument; (2) SB domain or activity performed; (3) type of exposure assessment; (3) data collection procedure; (4) statistical methods and confounding factors. For the assessment of cognitive function, the following aspects were examined: (1) cognitive function domain or domains; (2) instrument used; (3) data collection procedure; (4) association with the SB domain. Regarding cognitive domains, some terminologies were standardised in this manuscript due to heterogeneity across studies.

To improve clarity regarding the intended evaluation, studies assessing immediate and delayed memory operational terms were standardised as short‐term and long‐term memory theoretical terms. In addition, given the complexity of assessing a single domain without interference from others, some studies indicated that the instrument assessed more than one cognitive function, such as executive function and processing speed or working memory and short term memory.

## 7 Assessment of Risk of bias and Quality of Evidence

Two authors TRJS and EPD assessed the quality of the articles using two tools appropriate for different study designs, both developed by the same international centre for health evidence research, the Joanna Briggs Institute JBI [14]. The quality of included cohort and or case control studies was assessed using a checklist composed of 11 questions [15]. For cross sectional and analytical studies, a critical appraisal checklist composed of eight questions was used [16]. Both tools included response options of yes, no or unclear. For studies that presented both cross sectional and longitudinal analyses included in this review, the risk of bias was assessed separately for each study design according to the appropriate methodological tool.

Two evaluators TRJS and EPD independently used a Microsoft Excel spreadsheet 2024 to estimate study quality. The assessment considered three possible classifications for each item evaluated: low risk, unclear risk and high risk. The evaluators applied the following scoring criteria to determine study quality in cross sectional and cohort studies: low risk of bias if 70% of responses were scored as yes, unclear risk if 50%–69% of responses were scored as yes and high risk of bias if fewer than 50% of responses were scored as yes [17].

## 8 Results

**FIGURE 1:**
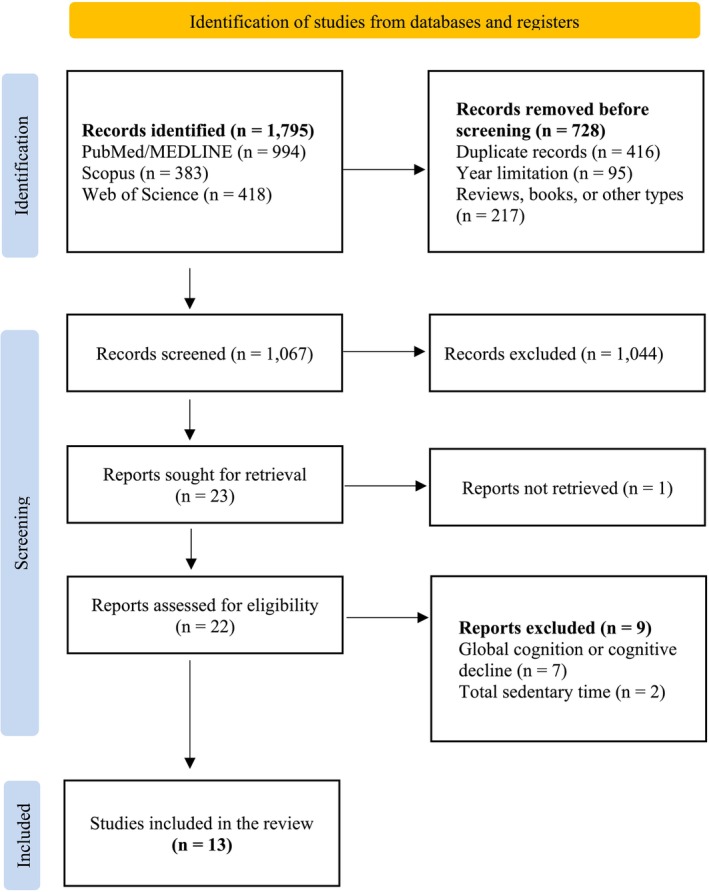
Flow diagram of the manuscript screening process.

The initial search yielded a total of 1795 manuscripts. For analysis, titles and abstracts were first screened. Subsequently, full text articles that potentially met the inclusion criteria were reviewed in detail. After these stages, 13 manuscripts that fulfilled the eligibility criteria were included (Figure 1). One study [18] appeared to meet the inclusion criteria based on title and abstract screening; however, the full text could not be accessed despite attempts to retrieve it from complementary databases and through contact with the authors. As a result, this record did not proceed to the full‐text eligibility assessment and was not included in the review.

## 9 Study Characteristics

Among the studies included in this systematic review, seven presented an exclusively cross sectional design, two adopted a cohort design and four combined cross sectional and longitudinal analyses within the same manuscript. The age range varied, with mean or median values between 51 and 90 years or older. In studies whose total sample included individuals below the age threshold defined for the review ≥ 60 years, only results stratified for the older adult subgroup were considered in the analyses. Accordingly, the final synthesis comprised data from 43 902 participants.

The specific objectives of the studies varied, mainly due to the inclusion of different outcome variables. Nevertheless, with regard to SB and cognitive function, all studies investigated the association between one or more SB domains and indicators of cognitive performance, encompassing executive function, language, memory and other relevant constructs (Table 1).

**TABLE 1:** Main characteristics of the included studies.

References	Study design	Age range (mean and SD/CI)	*n*	Study objective
Coelho et al. (2020) [19]	Cross‐sectional	74.6 (± 9.0)	75	To examine the relationship between sedentary time and cognitive ability using a robust assessment of cognitive abilities; To determine whether the association between sedentary time and executive function is different among individuals who engage in regular physical activity compared to those who are inactive.
Edwards and Loprinzi (2017) [20]	Cross‐sectional	69.9 (69.3–70.4)	2.472	Examine the independent association of SB and cognitive function in older adults, as well as whether or not physical activity attenuates this potential association.
Hu et al. (2025) [21]	Cohort (prospective)	70.8 (±5.7)	517	To investigate the association between mentally active SB and cognitive function in rural older Chinese across different levels of education.
Kesse‐Guyot et al. (2014) [22]	Cohort	65.6 (±4.5)	2.430	To examine the association between individual and clustered lifestyle behaviours and later cognitive functioning.
Kesse‐Guyot et al. (2012) [23]	Cross‐sectional and cohort	65.6 (±4.5)	2.179	To estimate the associations between different SBs and cognitive performance in healthy older adults.
Maasakkers et al. (2021) [24]	Cross‐sectional and cohort^a^	67 (±9.0)	6.687	Evaluate how subjective TV time and objectivelymeasured SB are associated with each other, but also how they are crosssectionally and longitudinally (4‐year follow‐up period) associated with global and domain‐specific cognitive outcomes in healthy older adults. Examine the reverse association between cognitive decline and future SB, we investigated the bidirectionality of the relation between SB and cognition.
Mellow et al. (2022) [25]	Cross‐sectional	65.6 (±3.0)	384	To understand how 24‐h time‐use composition is associated with cognitive function across a range of domains in healthy older adults and whether the level of recreational physical activity, amount of TV watching or the quality of sleep impact these potential associations.
Moreira et al. (2022) [26]	Cross‐sectional	61 (58–66)	6.505	To verify the association between SB and performance on cognitive function tests in middle‐aged and elderly adults.
Olanrewaju et al. (2020) [27]	Cross‐sectional and cohort (prospective)	63.5 (±9.2)	8.163	To explore the cross‐sectional associations between reported sedentary behaviours and cognitive function at baseline Wave 1 (sitting time) and Wave 3 (television viewing time); To explore the longitudinal associations between baseline sedentary behaviours and cognitive changes at 4‐year (Waves 1–3) and 2‐year follow‐up (Waves 3–4) in community dwelling adults 50 years and older, while accounting for well‐established socio‐economic, behavioural and health‐related confounders.
Palazuelos‐González et al. (2025) [28]	Cross‐sectional	51 (±8.8)^b^	11.087^b^	To examine the association between time spent in physical activities, SB and sleep with executive functioning and to estimate the influence of reallocating time from SB to other activities on executive functioning.
Rosenberg et al. (2015) [29]	Cross‐sectional	83.6 (±6.4)	307	Examined the relationships between objective and self‐reported sedentary time and health indicators among older adults residing in retirement communities.
Wingood et al. (2024) [30]	Cross‐sectional and cohort	≥ 65	2.244	To describe the types and amount of SB performed by older adults with and without impaired orientation, memory and executive function; To examine how participating in different types and amounts of SB at baseline impacts longitudinal changes in orientation, memory and executive function.
Zhou et al. (2022) [31]	Cross‐sectional	≥ 80	852	I Identify distinct subgroups of oldest old adults based on six domains of SB (watching TV, using a computer/tablet, talking to friends or family members, doing hobby or other activities, transportation and resting/napping); III Compare health‐related outcomes across identified subgroups, using the National Health and Aging Trends Study (NHATS) dataset.

Abbreviations: CI: confidence interval; SD: standard deviation.

^a^ The assessment of methodological quality considered only the cross‐sectional design, as the study used specific SB domains rather than total sedentary time alone.

^b^ Results were analysed exclusively in the age stratum of 60 years or older.

Additionally, a wide diversity was observed in terms of the countries in which the studies were conducted. Four were carried out in the United States [20, 29, 30, 31], two in Ireland [24, 27], two in France [22, 23], one in the Netherlands [28], one in China [21], one in Australia [25], one in Canada [19] and one in Brazil [26].

## 10 Assessment of SB

Most studies (77%; 10 of 13) used only subjective methods to assess SB through standardised questionnaires or single questions [19, 20, 21, 22, 23, 26, 27, 28, 30, 31]. In contrast, 23% of the studies (3 of 13) combined objective measures using accelerometry with questionnaires [24, 25, 29]. However, in these studies that employed accelerometers, only the subjective data were analysed due to the need to assess the context of SB.

The most frequently investigated domain was television viewing, reported in 85% of the studies (11 of 13) [20, 22, 23, 24, 25, 26, 27, 28, 29, 30, 31]. In addition, 62% (8 of 13) examined computer, tablet or Internet use [19, 20, 23, 25, 28, 29, 30, 31]. Other less frequent domains included reading, hobbies, games and sedentary time spent driving or as a passenger in a motor vehicle.

All studies (100%) administered questionnaires in person with interviewers, demonstrating methodological standardisation in this aspect. In studies that included accelerometry (23%), devices were worn for periods ranging from 6 to 7 days [24, 25, 29].

Regarding statistical methods, considerable analytical heterogeneity was observed among the included studies. Linear regression, either simple or multiple, was the predominant approach, used in 54% of the studies (7 of 13) [19, 20, 24, 26, 27, 28, 31], although this method has limitations in modelling complex relationships between behavioural variables and cognitive domains. Other studies employed analysis of covariance (ANCOVA) [22, 23, 25] or linear mixed effects models [21, 29], the latter being more appropriate for the analysis of repeated measures and for accounting for within‐subject dependence over time.

Beyond these approaches, some studies applied more sophisticated statistical methods, including isotemporal substitution models [28], which allow estimation of counterfactual effects by reallocating time between different behaviours; structural equation modelling [19], which enables the assessment of latent relationships between cognitive constructs and components of SB and more recent analyses based on *χ* ^2^ tests with Bonferroni correction and discrete time competing risk models [30], thereby expanding the ability to capture associations in scenarios with multiple competing outcomes (Table 2).

**TABLE 2:** Characteristics of SB assessment and analytical approaches used.

References	Measurement instrument	SB domain or activity performed	Type of exposure assessment	Data collection procedure	Statistical methods
Coelho et al. (2020) [19]	LASA‐SB (Longitudinal Ageing Study Amsterdam—SB)	Watching television, napping, reading, listening to music, socialising and engaging in hobbies.	Subjective	Interviewer‐administered questionnaire (in person).	Correlation analyses and multiple linear regression
Edwards and Loprinzi (2017) [20]	Self‐reported data from NHANES	Watching television or videos or using a computer outside of work.	Subjective	Interviewer‐administered questionnaire (in person).	Multivariable linear regression.
Hu et al. (2025) [21]	Single question	Card games or mahjong; chess or checkers; calligraphy, painting, reading or writing during leisure time.	Subjective	Interviewer‐administered questionnaire (in person).	Linear mixed‐effects models.
Kesse‐Guyot et al. (2014) [22]	Modifiable Activity Questionnaire (MAQ)—French version	SB was assessed as the average daily time spent watching television at home.	Subjective	Interviewer‐administered questionnaire (in person).	Analysis of covariance (ANCOVA); structural equation modelling (SEM); principal component analysis (PCA)
Kesse‐Guyot et al. (2012) [23]	MAQ Questionnaire (Modifiable Activity Questionnaire)—French version	Computer use, television viewing and reading	Subjective	Interviewer‐administered questionnaire (in person).	Analysis of covariance (ANCOVA).
Maasakkers et al. (2021) [24]	GENEActiv accelerometer plus a single question on television viewing time	Total sedentary time and time spent watching television.	Objective and subjective	Accelerometer (wrist‐worn for 7 days). Interviewer‐administered questionnaire (in person).	Longitudinal mixed models (bidirectional associations).
Mellow et al. (2022) [25]	Triaxial accelerometer (model not specified) and multimedia activity recall for children and adults (MARCA)	Television viewing and total SB.	Objective and subjective	Accelerometer (waist‐worn for 7 days); Interviewer‐administered questionnaire (in person).	Multiple linear regression models; Analysis of covariance (ANCOVA).
Moreira et al. (2022) [26]	Single question on total SB and screen time	Total sedentary time and screen time (television, video games, computers, tablets, smartphones, etc.).	Subjective	Interviewer‐administered questionnaire (in person).	Linear regression
Olanrewaju et al. (2020) [27]	International Physical Activity Questionnaire (IPAQ) and a single question on television viewing (TILDA data)	Total sitting time and time spent watching television on weekdays.	Subjective	Interviewer‐administered questionnaire (in person).	Linear regression analyses (cross‐sectional and longitudinal).
Palazuelos‐González at al (2025) [28]	Self‐reported	Time spent watching television.	Subjective	Interviewer‐administered questionnaire (in person).	Multivariable linear regression and isotemporal substitution analysis.
Rosenberg et al. (2015) [29]	GT3X accelerometer and SB Questionnaire (SBQ)—modified version	Television viewing, computer or internet use, reading, socialising, transportation, hobbies and group activities. For analysis, SB was aggregated as total sedentary time, but television viewing was analysed separately.	Objective and subjective	Accelerometer (waist‐worn for 6 days); Interviewer‐administered questionnaire (in person).	Linear mixed‐effects models.
Wingood et al. (2024) [30]	Self‐reported	Watching television, computer use, socialising, hobbies, transportation, eating and resting.	Subjective	Interviewer‐administered questionnaire (in person).	*χ* ^2^ tests and *t*‐tests with Bonferroni correction (Objective 1); Discrete‐time competing risk models (Objective 2)
Zhou et al. (2022) [31]	Subjective questionnaire (SB Questionnaire—SBQ)	Television viewing; computer or tablet use; socialising with friends or family; engaging in hobbies; transportation.	Subjective	Interviewer‐administered questionnaire (in person).	Latent profile analysis (LPA); Linear and logistic regression analyses.

Among the cognitive domains most frequently investigated, memory was the most commonly assessed overall, accounting for 76.9% (10 of 13) of citations across studies. However, the included studies associated SB with one or more memory subsystems. Accordingly, there was variation in the examination of this domain, including assessments of short‐term and long‐term memory, as well as semantic and phonemic memory.

Executive function was the second most frequently evaluated domain, with a prevalence of 53.8% (7 of 13). One study assessed processing speed as an extension of executive function [20]. The remaining domains were investigated less frequently, with proportions ranging from 15.3% (2 of 13) to 7.7% (1 of 13).

In the analysis of the included studies (*n* = 13), a predominance of instruments aimed at assessing memory and executive function was identified. Among the most frequently used tests, the 10 word list stands out, applied in different versions and contexts, including the CERAD protocol, as a standalone measure or integrated into cognitive batteries, and employed in several studies [19, 20, 21, 24, 26, 27, 30, 31]. This instrument was widely used to measure short term and long term memory, constituting the most frequent method for the assessment of these cognitive domains.

Another group of widely used instruments consisted of verbal fluency tests, both semantic, such as category based tasks including animal naming and phonemic, based on letter generation, employed in several studies [22, 23, 24, 26, 27]. These tests were relevant for measuring semantic and phonemic verbal memory and language related components, including lexical access, retrieval speed and semantic organisation. Trail Making Tests A and B were also used in several studies [22, 23, 26, 29], particularly, to assess cognitive flexibility, sustained attention, processing speed and components of executive function.

In addition, other instruments were used less frequently but remain relevant for cognitive assessment. These included forward and backward Digit Span tests, used to measure working memory and attention [22, 23]; the Digit Symbol Substitution Test (DSST), applied by Edwards and Loprinzi [20] to assess processing speed and aspects of executive function and the Ruff Figural Fluency Test (RFFT), employed by Maasakkers et al. [24] to measure non‐verbal fluency and cognitive flexibility. More comprehensive cognitive batteries were also identified, such as the CERAD [26], the CANTAB [25], the Mini‐Mental State Examination (MMSE) [20] and the TICS‐10 [21], designed to assess global cognition and multiple cognitive domains.

## 11 Associations With SB

Time spent watching television was the SB domain most consistently associated with poorer cognitive outcomes, reported in 61.5% of the included studies (8 of 13) [19, 20, 22, 24, 26, 27, 28, 31]. However, this association was not uniform. Two studies by Rosenberg et al. [29] and Mellow et al. [25] did not identify a relationship between television viewing time and cognitive performance across the different domains evaluated. In contrast, the study by Wingood et al. [30] was the only one to demonstrate a potential protective effect of television viewing time on memory performance.

Computer or internet use showed a positive association with better performance in memory and executive functions in 23% of the studies (3 of 13) [19, 23, 26]. In addition, other studies observed that cognitively stimulating sedentary activities, such as card games, hobbies, reading and other behaviours considered mentally active, were associated with better performance in specific domains, including orientation, attention and short‐term and long‐term memory. In contrast, SBs classified as mentally passive were associated with poorer outcomes in several cognitive subdomains [21, 31].

All included studies adjusted their analyses for sociodemographic variables, with particular emphasis on age, sex and educational level, which were the most frequently applied adjustments. Other commonly considered factors, reported in more than 70% of the studies, included body mass index, physical activity level, smoking, alcohol consumption, presence of chronic diseases, depressive symptoms and sleep quality (Table 3).

**TABLE 3:** Characteristics of cognitive function assessment and corresponding associations with SB.

References	Cognitive function domain(s) assessed	Measurement instrument or test	Association with the SB domain	Confounding factors
Coelho et al. (2020) [19]	– Executive function; – Short‐ and long‐term memory.	– BRIEF‐A, Stroop, Tower of Hanoi, LEGO/Mega Blocks, Snap; – Logical memory.	Greater time spent watching television, napping and engaging in sedentary hobbies was associated with poorer executive function, with only napping being associated with memory; other forms of SB (such as attending church and driving) did not impact cognition.	Age, level of physical activity and sex.
Edwards and Loprinzi (2017) [20]	– Executive function and processing speed.	Digit symbol substitution test (DSST)	SB (television, videos or computer use) of at least 5 h per day was associated with poorer performance; however, the association lost statistical significance after adjustment for physical activity.	Age, sex, body mass index, race, smoking, physical activity and coronary artery disease.
Hu et al. (2025) [21]	– Orientation; – Attention; – Short‐ and long‐term memory; – Global cognition.	Telephone Interview for Cognitive Status (TICS‐10); – Spatial orientation (current day, month and year); – Serial subtraction (serial 7 s); – 10‐word list; – Sum of all tests.	Mentally active SB (card games, time spent playing mahjong and or reading or painting) was positively associated with cognitive subdomains, with stronger associations observed among illiterate individuals.	Age, sex, educational level, income, marital status, sleep, physical activity, body mass index, alcohol consumption and smoking.
Kesse‐Guyot et al. (2014) [22]	– Working memory or short‐term memory; – Mental flexibility; – Long‐term memory; – Semantic verbal memory; – Phonemic verbal memory.	– Forward and backward Digit Span; – Trail Making Test (TMT); – RI‐48 test; – Animal naming; – Phonemic fluency (letter P).	SB (watching television for more than 1 h per day) was negatively associated with verbal memory in the unadjusted model; however, this association lost statistical significance after adjustment.	Age, sex, educational level, body mass index, physical activity, depressive symptoms and chronic diseases.
Kesse‐Guyot et al. (2012) [23]	– Phonemic verbal memory; – Semantic verbal memory; – Long‐term memory; – Working memory or short‐term memory; – Mental flexibility.	– Phonemic fluency test (letter P); – Semantic fluency test (animals); – RI‐48 test (free and cued recall test); – Forward and backward Digit Span; – Trail Making Test (TMT Part B).	Computer use was positively associated with verbal memory and executive function, even after 6 years, whereas television viewing was negatively associated with executive function in cross‐sectional analyses.	Age, sex, educational level, marital status, occupation, alcohol consumption, smoking, physical activity, depression, antioxidant supplementation and body mass index.
Maasakkers et al. (2021) [24]	– Short‐ and long‐term memory; – Semantic verbal memory; – Global cognition.	– 10‐word list; – Animal naming; – Mini‐Mental State Examination (MMSE).	A weak negative correlation was observed between television viewing time and memory, which was explained by confounding factors. No longitudinal associations were found between television viewing time and the outcome.	Age, sex, educational level, depression, body mass index, comorbidities, moderate to vigorous physical activity, sleep, mobility and self‐rated health.
Mellow et al. (2022) [25]	– Short‐term memory; – Long‐term memory; – Executive function; – Processing speed.	Cambridge Automated Neuropsychological Test Automated Battery (CANTAB); – Word list; – Word list and Paired Associates Learning; – Multitasking test; – Reaction time.	The study found no significant effect of television viewing time on cognitive function, including memory, executive function and processing speed.	Age, sex, educational level, place of data collection, smoking status, sleep and recreational physical activity.
Moreira et al. (2022) [26]	– Short‐ and long‐term memory; – Language; – Executive function.	CERAD test battery; – 10‐word list; – Semantic fluency test (flora) and phonemic fluency test (letter A); – Trail Making Test (Part B).	Greater sedentary time in occupational screen use was positively associated with performance on memory, language and executive function tests, with variations between men and women.	Age, sex, educational level, race or ethnicity, income, depression, body mass index, physical activity, smoking and alcohol consumption.
Olanrewaju et al. (2020) [27]	– Short‐ and long‐term memory; – Semantic verbal memory.	– 10‐word list; – Animal naming.	Greater time spent watching television was associated with poorer performance in verbal memory and fluency, particularly among adults aged 65 years or older who watched television for at least 3.5 h per day after 2 years.	Age, sex, social class, employment status, physical activity, loneliness, obesity, depression, disabilities and chronic diseases.
Palazuelos‐González et al. (2025) [28]	– Executive function	– Ruff figural fluency test (RFFT)	Time spent watching television was negatively associated with executive function; replacing this time with physical activities (sports, work and leisure) was associated with improved executive function.	Age, sex, educational level, occupation, depression, anxiety, chronic diseases, body mass index, smoking, alcohol consumption, diet and social contacts
Rosenberg et al. (2015) [29]	– Visual search and perceptual speed; – Executive function.	– Trail Making Test (Part A); – Trail Making Test (Part B).	Weak association: self‐reported SB aggregated across contexts was related to better performance on Trail Making Test Part A, whereas television viewing time showed no significant association.	Age, sex, educational level, marital status, fear of falling, pain, stress, depression, sleep, physical activity and chronic diseases.
Wingood et al. (2024) [30]	– Orientation; – Short‐ and long‐term memory; – Executive function.	– Spatial orientation (current day, month and year); – 10‐word list; – Clock drawing test.	Older adults who engage more frequently in cognitively active SB (computer use and hobbies) show a lower risk of impairment in orientation and memory, whereas passive SB, except for television viewing for more than 3 h per day in longitudinal analyses, do not provide cognitive protection.	Age, sociodemographic characteristics, self‐rated health, physical function and educational level.
Zhou et al. (2022) [31]	– Short‐term memory.	– 10‐word list.	Mentally passive SB (television viewing and napping) were associated with poorer cognition, particularly short‐term memory, whereas mentally active behaviours (hobbies and computer use) were associated with better cognitive performance.	Age, sex, race, educational level and marital status.

## 12 Risk of Bias

The assessment of risk of bias in cross sectional studies (*n* = 11) showed variation between low, moderate and high risk (Appendix B). Approximately 45% (5 of 11) of the studies were classified as having low risk of bias [23, 26, 27, 28, 29], suggesting methodological consistency across different evaluated domains.

Another 45% (5 of 11) presented moderate risk [19, 20, 25, 30, 31], mainly due to limitations related to the clarity of inclusion criteria, characterisation of the sample and context, validity and reliability of exposure measures and definition of the diagnostic criteria used. In contrast, the study by Maasakkers et al. [24] was classified as having high risk of bias due to relevant shortcomings across the domains.

Overall, the domains most consistently well evaluated were those related to control of confounding factors D6, validity and reliability of outcomes D7 and adequacy of statistical analysis D8, in which most studies were classified as having low risk of bias. Conversely, the weakest points were concentrated in the domains of inclusion criteria D1, description of sample and context D2, validity and reliability of exposure D3 and objective criteria for the condition D4, where a higher frequency of moderate and high risk was observed. Minor inconsistencies were also noted in the domain of identification of confounding factors D5 (Appendix C).

Taken together, the results indicate that although most studies present satisfactory methodological quality, relevant limitations persist, particularly, those related to clarity and precision in the measurement of different SB domains and in the assessment of cognitive conditions. These methodological weaknesses may compromise the robustness of effect estimates and influence the interpretation of the observed associations between SB and cognitive function. The assessment of risk of bias in cohort studies demonstrated that all were classified as having low risk of bias [21, 22, 23, 24, 27, 30] (Appendix D), indicating adequate methodological consistency across the main evaluated domains.

Overall, the domains with the best performance were group comparability (D1), identification of and strategies to control confounding factors (D4 and D5) and adequacy of statistical analysis (D11), all of which were predominantly rated as having low risk of bias. In contrast, the most critical points were identified in the domains of validity and reliability of exposure measurement (D3), validity and reliability of outcome measurement (D7) and completeness of follow up (D9), which showed a higher proportion of moderate risk (Appendix E). Thus, although the reviewed cohort studies generally present satisfactory methodological quality, there remains a need for greater rigour in the measurement of exposures and outcomes, as well as clearer reporting of follow up procedures and associated losses.

## 13 Discussion

This study systematically analysed the impact of different contexts of SB, such as watching television, use of electronic devices, reading or playing games, on different cognitive domains in older adults. The findings of this review demonstrate that the impact of SB on cognitive function in older adults strongly depends on the type of activity performed during sedentary time.

It was observed that mentally passive SB, such as watching television and napping, tends to be negatively associated with cognitive outcomes, particularly, in domains related to memory and executive function. In contrast, mentally active behaviours, including computer use, reading and other cognitively stimulating activities, showed positive associations with cognitive performance or with a smaller decline in performance over time.

Time spent watching television is one of the most prevalent SB domains in the context of leisure, especially among older adults. The study by Bertuol et al. [32] showed that between 2006 and 2016 older adults in Brazil presented nearly twice the prevalence of this behaviour compared with adults, largely due to resistance to other technologies. Although television viewing has been negatively associated with several physical health determinants because of physiological factors [33], its relationship with brain functioning is complex due to multifactorial influences of biological, psychological and social nature [34].

Consistent with the findings of the present review, Xu et al. [35] observed that time spent watching television was negatively associated with long‐term cognitive function, particularly, among individuals with central nervous system diseases such as dementia, stroke and Parkinson disease. Conversely, Cegolon and Jenkins [36] demonstrated that cognitively stimulating activities, such as computer use, games, reading and other mentally active tasks, were associated with preservation of cognitive performance, in line with the theoretical principle of use it or lose it, according to which continuous engagement in mental activities contributes to the maintenance and strengthening of cognitive functions.

This association is multi‐faceted. From a neurophysiological perspective, excessive time spent on certain screens compromises grey matter volume, which is responsible for information processing [37, 38]. However, television screen time differs from computer use in terms of cognitive stimulation, as the former is less demanding of working memory, planning and problem solving, directly influencing cortical activation, synaptic stimulation and the release of neurotrophic factors that are essential for dendritic maintenance [39].

Additionally, from psychological and social perspectives, mentally passive behaviours may lead to low motivation, depressive symptoms, social isolation and reduced community engagement [40, 41, 42]. Thus, cognition, as a central component of brain activity, both influences and is influenced by mental and social factors and is shaped according to the level of neurobiological stimulation that allows the preservation of cognitive subdomains [43].

Nevertheless, cognitive abilities, although interacting in complex ways and functioning in an integrated and dynamic manner, also present domain‐specific functioning and perform distinct tasks [44]. For this reason, some outcomes in the studies included in this systematic review reveal distinct interactions with different SB domains, depending on the context in which they occur and the activity performed [21, 22, 30].

Tasks that rely on executive function involve planning, decision making, cognitive flexibility and execution of daily activities [45]. Accordingly, this ability is related to physical activity, which, by engaging neurobiological and behavioural mechanisms inherent to bodily movement, positively impacts the brain system [46]. This relationship explains the inclusion of physical activity as a confounding factor in some studies.

In contrast, language, memory and orientation require not only factors such as educational level, sex and age, which influence performance on many cognitive assessment tests, but also individual repertoire and participation in activities that stimulate these abilities at different levels [47]. This further supports the argument regarding the complexity of SB types and their multiple facets.

Although this review focused on the context and type of SB, time spent in these activities is also relevant. Evidence suggests that longer durations of mentally passive behaviours, especially television viewing, are more consistently associated with poorer cognitive outcomes, while shorter or moderate engagement shows weaker associations [48, 49]. However, heterogeneity in time measurement and categorisation across studies prevented direct comparisons and dose–response analysis. Thus, findings reflect qualitative differences between SB domains rather than precise exposure thresholds.

Regarding risk of bias, an initial individual assessment of each manuscript was conducted, followed by an integrated analysis of the domains most susceptible to methodological weaknesses. A large proportion of studies presented relevant limitations in the measurement of SB and in the definition of cognitive conditions, as well as insufficient transparency regarding inclusion and exclusion criteria. In longitudinal studies, gaps were also identified in the description and handling of follow up losses, which may compromise the internal validity of the estimates.

The use of validated instruments with adequate psychometric properties reflects greater methodological rigour [50]. In order to minimise estimation errors related to self‐reported measures or cognitive tests, the instruments used need to be tested and compared with others to confirm their reliability and accuracy [51]. Likewise, studies must clearly report sample characteristics to avoid misinterpretations and potential distortions.

In addition, this review revealed marked heterogeneity regarding the countries of origin of the studies. It is well recognized that daily behaviours and habitual practices vary substantially across different cultural contexts [52]. Thus, although all manuscripts included older adult populations, the ways in which individuals engage in SB and cognitively active behaviours reflect specific sociocultural characteristics, such as gender norms, educational levels and access to digital technologies, which directly influence the relationship between SB and cognitive performance [53]. These contextual factors should be carefully considered when interpreting and comparing the observed associations.

In light of this scenario, the findings of this study have important scientific implications. They highlight the need for strategies aimed at reducing time spent in passive forms of SB, favouring their substitution with cognitively stimulating activities. Furthermore, they reveal gaps that create opportunities for the development of digital and leisure based interventions focused on active mental engagement. Finally, they underscore the urgency of studies with greater methodological robustness, particularly longitudinal and experimental designs, to elucidate causal relationships between SB and cognitive function.

An important limitation of this review is that the search strategy focused on SB terms, which may have led to the exclusion of studies examining cognitively engaging leisure activities that are not explicitly framed in terms of sitting time. Consequently, the findings should be interpreted within the context of SB research rather than cognitively stimulating activities per se. Another limitation is that SB domains were predominantly assessed through self‐report, which may be affected by recall bias, particularly for habitual and prolonged activities, potentially influencing the observed associations.

## 14 Conclusion

The findings of this systematic review indicate that SB is consistently associated with cognitive function in older adults, but this relationship strongly depends on the type of activity performed during sedentary time. Time spent watching television was the most extensively investigated domain and showed a negative association with cognition in a large proportion of studies, particularly with memory and executive function, suggesting that mentally passive SB may contribute to the decline of certain cognitive functions during ageing.

In contrast, sedentary activities considered mentally active, such as computer use, reading, card games and hobbies, were associated with better cognitive performance, indicating that cognitive engagement during sitting time may play a protective role in brain function. Despite the predominance of subjective methods for measuring SB, the consistency of findings across different countries and study designs reinforces the relevance of the context in which SB occurs, rather than focusing solely on total sedentary time. However, methodological heterogeneity and limitations in the validity and precision of exposure measures still warrant caution in the interpretation of the findings.

Overall, the results support the hypothesis that not all SB is equally harmful to cognition. Engagement in cognitively stimulating activities, even while sitting, may mitigate some of the negative effects associated with prolonged sedentary time. Therefore, health promotion strategies aimed at healthy ageing should not only seek to reduce total exposure to SB but also encourage the replacement of passive SB with cognitively active activities.

## Funding

The authors have nothing to report.

## Ethics Statement

The authors have nothing to report.

## Conflicts of Interest

The authors declare no conflicts of interest.

## Data Availability

Data sharing not applicable to this article as no datasets were generated or analysed during the current study.

## Appendix A See Table A1

**TABLE A1:** Search strategy for study selection.

PubMed/Medline	
#1	(‘Aged’[Mesh]) OR (senior) OR (old person) OR (older age) OR (older person) OR (older adult) OR (elderly) OR (elderly population)
#2	(‘Sedentary Behaviour’[Mesh]) OR (Sedentary Lifestyle) OR (sitting time) OR (sedentary time)
#3	(‘Cognition’[Mesh]) OR (performance cognitive) OR (cognitive function)
#4	#1 AND #2 AND #3

Scopus
TITLE‐ABS‐KEY((‘Older Adults’ OR ‘Aged’ OR ‘elderly’ OR ‘sênior’ OR ‘older age’ OR ‘old person’ OR ‘elderly population’ OR ‘older person’) AND (‘Sedentary Behaviour’ OR ‘Sitting time’ OR ‘sedentary time’ OR ‘Sedentary Lifestyle’) AND (‘Cogniton’ OR ‘Cognitive Function’ OR ‘Performance Cognitive’))

Web of Science
‘Older Adults’ OR ‘Aged’ OR ‘Elderly’ OR ‘Senior’ OR ‘Older Age’ OR ‘Old Person’ OR ‘Elderly Population’ OR ‘Older Person’ AND ‘Sedentary Behaviour’ OR ‘Sitting Time’ OR ‘Sedentary Time’ OR ‘Sedentary Lifestyle’ AND ‘Cognition’ OR ‘Cognitive Function’ OR ‘Cognitive Performance’

Abbreviation: tw, text word.

## Appendix B See Table B1

**TABLE B1:** Risk of bias for cross‐sectional studies.

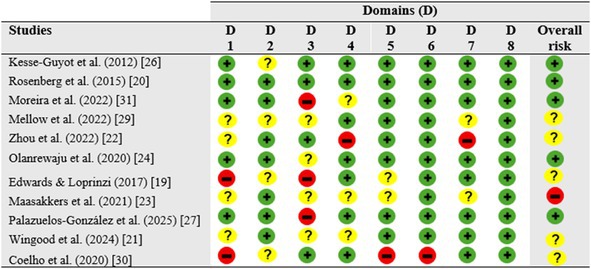

Abbreviations: D1: clear inclusion criteria; D2: description of sample and context; D3: validity and reliability of exposure; D4: objective criteria for the condition; D5: identification of confounding factors; D6: control of confounding factors; D7: validity and reliability of outcomes; D8: adequate statistical analysis.

Low:  Unclear: High:

## Appendix C See Table C1

**TABLE C1:** Risk of bias by domain assessed in cross‐sectional studies.

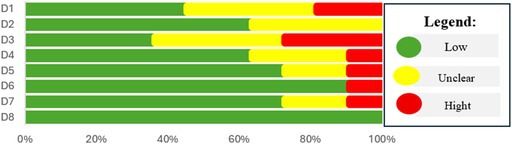

## Appendix D See Table D1

**TABLE D1:** Risk of bias for cohort studies.

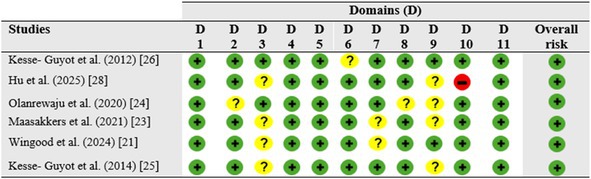

Abbreviations: D1: similarity of groups and recruitment from the same population; D2: uniform measurement of exposure for all groups; D3: validity and reliability of exposure measurement; D4: identification of confounding factors; D5: strategies to control confounding factors; D6: absence of the outcome at the start of the study or exposure; D7: validity and reliability of outcome measurement; D8: adequate follow‐up time for outcome occurrence; D9: completeness of follow‐up and description of losses; D10: strategies to address incomplete follow‐up; D11: adequate statistical analysis.

Low:  Unclear:  High:

## Appendix E See Table E1

**TABLE E1:** Risk of bias by domain assessed in cohort studies.

## References

[1] D. D. Farhud , “Impact of Lifestyle on Health,” Iranian Journal of Public Health 44, no. 11 (2015): 1442–1444. 26744700 PMC4703222

[2] V. Barbaccia , L. Bravi , F. Murmura , E. Savelli , and E. Viganò , “Mature and Older Adults' Perception of Active Ageing and the Need for Supporting Services: Insights From a Qualitative Study,” International Journal of Environmental Research and Public Health 19, no. 13 (2022): 7660. 35805320 10.3390/ijerph19137660 PMC9265376

[3] T. Sahinoz and S. Sahinoz , “Investigation of Healthy Living Strategies in Elderly Who Achieved to Live Long and Healthy,” Pakistan Journal of Medical Sciences 36, no. 3 (2020): 371–375. 32292436 10.12669/pjms.36.3.1838 PMC7150393

[4] S. Kalyoncuo and P. T. Kartin , “The Relationship Between Active Aging and Healthy Lifestyle Behaviors of Individuals Aged 65 Years and Older: A Cross‐Sectional Study,” Geriatric Nursing 61 (2025): 316–323. 39577379 10.1016/j.gerinurse.2024.10.063

[5] B. G. G. da Costa , J. P. Chaput , and K. S. Silva , “The Two Sides of Sedentary Behavior,” Journal of Physical Education 33 (2022): e3312.

[6] D. K. Jang , M. Park , and Y. H. Kim , “Sociodemographic, Behavioural, and Health Factors Associated With Sedentary Behaviour in Community‐Dwelling Older Adults: A Nationwide Cross‐Sectional Study,” Journal of Clinical Medicine 12, no. 15 (2023): 5005. 37568405 10.3390/jcm12155005 PMC10419473

[7] M. E. Costa , L. M. Cândido , N. C. P. de Avelar , and A. L. Danielewicz , “How Much Time of Sedentary Behavior Is Associated With Depressive Symptoms in Community‐Dwelling Older Adults in Southern Brazil?,” Geriatric Nursing 50 (2023): 25–30. 36640515 10.1016/j.gerinurse.2022.12.014

[8] X. Y. Cai , G. P. Qian , F. Wang , M. Y. Zhang , Y. J. Da , and J. H. Liang , “Association Between Sedentary Behavior and Risk of Cognitive Decline or Mild Cognitive Impairment Among the Elderly: A Systematic Review and Meta‐Analysis,” Frontiers in Neuroscience 17 (2023): 1221990. 37600015 10.3389/fnins.2023.1221990 PMC10436513

[9] K. Dillon , A. Morava , H. Prapavessis , L. Grigsby‐Duffy , A. Novic , and P. A. Gardiner , “Total Sedentary Time and Cognitive Function in Middle‐Aged and Older Adults: A Systematic Review and Meta‐Analysis,” Sports Medicine—Open 8, no. 1 (2022): 127. 36224459 10.1186/s40798-022-00507-x PMC9556686

[10] P. D. Harvey , “Domains of Cognition and Their Assessment,” Dialogues in Clinical Neuroscience 21, no. 3 (2019): 227–237. 31749647 10.31887/DCNS.2019.21.3/pharvey PMC6829170

[11] D. A. Raichlen , Y. C. Klimentidis , M. K. Sayre , et al., “Leisure‐Time Sedentary Behaviors Are Differentially Associated With All‐Cause Dementia Regardless of Engagement in Physical Activity,” National Academy of Sciences of the United States of America 119, no. 35 (2022): e2206931119. 10.1073/pnas.2206931119 PMC9436362 35994664

[12] M. J. Page , J. E. McKenzie , P. M. Bossuyt , et al., “The PRISMA 2020 Statement: An Updated Guideline for Reporting Systematic Reviews,” March 29, 2021. Cited January 7, 2026, https://www.bmj.com/content/372/bmj.n71. 10.1186/s13643-021-01626-4 PMC8008539 33781348

[13] Brasil , “Lei n^o^ 10.741, de 1^o^ de outubro de 2003. Sect. 1, 10741 Oct 3, 2003,” https://www.planalto.gov.br/ccivil_03/leis/2003/l10.741.htm.

[14] Joanna Briggs Institute , “JBI Critical Appraisal Tools | JBI,” 2017, Cited September 20, 2025, https://jbi.global/critical‐appraisal‐tools.

[15] Joanna Briggs Institute , “Checklist for Analytical Cross Sectional Studies,” 2020, Cited January 7, 2026, https://view.officeapps.live.com/op/view.aspx?src=https%3A%2F%2Fjbi.global%2Fsites%2Fdefault%2Ffiles%2F2021‐10%2FChecklist_for_Analytical_Cross_Sectional_Studies.docx&wdOrigin=BROWSELINK.

[16] Joanna Briggs Institute , “Checklist for Cohort Studies,” 2020, Cited January 7, 2026, https://view.officeapps.live.com/op/view.aspx?src=https%3A%2F%2Fjbi.global%2Fsites%2Fdefault%2Ffiles%2F2021‐10%2FChecklist_for_Cohort_Studies.docx&wdOrigin=BROWSELINK.

[17] C. M. Goplen , W. Verbeek , S. H. Kang , et al., “Preoperative Opioid Use Is Associated With Worse Patient Outcomes After Total Joint Arthroplasty: A Systematic Review and Meta‐Analysis,” BMC Musculoskeletal Disorders 20, no. 1 (2019): 234. 31103029 10.1186/s12891-019-2619-8 PMC6525974

[18] K. Bakrania , C. L. Edwardson , K. Khunti , S. Bandelow , M. J. Davies , and T. Yates , “Associations Between Sedentary Behaviors and Cognitive Function: Cross‐Sectional and Prospective Findings From the UK Biobank,” American Journal of Epidemiology 187, no. 3 (2018): 441–454. 28992036 10.1093/aje/kwx273

[19] L. Coelho , K. Hauck , K. McKenzie , et al., “The Association Between Sedentary Behavior and Cognitive Ability in Older Adults,” Aging Clinical and Experimental Research 32, no. 11 (2020): 2339–2347. 31898168 10.1007/s40520-019-01460-8

[20] M. K. Edwards and P. D. Loprinzi , “The Association Between Sedentary Behavior and Cognitive Function Among Older Adults May be Attenuated With Adequate Physical Activity,” Journal of Physical Activity & Health 14, no. 1 (2017): 52–58. 27775470 10.1123/jpah.2016-0313

[21] J. Hu , Q. Deng , C. Yong , et al., “The Relationship Between Mentally Active Sedentary Behavior and Cognitive Function Across Different Educational Levels,” Dementia and Geriatric Cognitive Disorders 54, no. 1 (2025): 1–9. 38964292 10.1159/000539863

[22] E. Kesse‐Guyot , V. A. Andreeva , C. Lassale , S. Hercberg , and P. Galan , “Clustering of Midlife Lifestyle Behaviors and Subsequent Cognitive Function: A Longitudinal Study,” American Journal of Public Health 104, no. 11 (2014): e170–e177. 25211733 10.2105/AJPH.2014.302121 PMC4202965

[23] E. Kesse‐Guyot , H. Charreire , V. A. Andreeva , et al., “Cross‐Sectional and Longitudinal Associations of Different Sedentary Behaviors With Cognitive Performance in Older Adults,” PLoS One 7, no. 10 (2012): e47831. 23082222 10.1371/journal.pone.0047831 PMC3474738

[24] C. M. Maasakkers , J. A. H. R. Claassen , S. Scarlett , et al., “Is There a Bidirectional Association Between Sedentary Behaviour and Cognitive Decline in Older Adults? Findings From the Irish Longitudinal Study on Ageing,” Preventive Medicine Reports 23 (2021): 101423. 34258171 10.1016/j.pmedr.2021.101423 PMC8259404

[25] M. L. Mellow , D. Dumuid , A. T. Wade , et al., “Twenty‐Four‐Hour Time‐Use Composition and Cognitive Function in Older Adults: Cross‐Sectional Findings of the ACTIVate Study,” Frontiers in Human Neuroscience 16 (2022): 1051793. 36504624 10.3389/fnhum.2022.1051793 PMC9729737

[26] P. d. A. Moreira , S. M. A. de Matos , F. J. G. Pitanga , et al., “Association Between Sedentary Behavior and Cognitive Performance in Middle‐Aged and Elderly Adults: Cross‐Sectional Results From ELSA‐Brasil,” International Journal of Environmental Research and Public Health 19, no. 21 (2022): 14234. 36361115 10.3390/ijerph192114234 PMC9654160

[27] O. Olanrewaju , A. Koyanagi , M. Tully , N. Veronese , and L. Smith , “Sedentary Behaviours and Cognitive Function Among Community Dwelling Adults Aged 50+ Years: Results From the Irish Longitudinal Study of Ageing,” Mental Health and Physical Activity 19 (2020): 100344.

[28] R. Palazuelos‐González , R. C. Oude Voshaar , S. la Bastide‐van Gemert , and N. Smidt , “Time Spent in Physical Activities, TV Watching and Sleep and Its Association With Executive Functioning in Middle Age and Older Adults: An Isotemporal Substitution Analysis,” Mental Health and Physical Activity 28 (2025): 100668.

[29] D. E. Rosenberg , J. Bellettiere , P. A. Gardiner , V. N. Villarreal , K. Crist , and J. Kerr , “Independent Associations Between Sedentary Behaviors and Mental, Cognitive, Physical, and Functional Health Among Older Adults in Retirement Communities,” Journals of Gerontology. Series A, Biological Sciences and Medical Sciences 71, no. 1 (2015): 78–83. 26273024 10.1093/gerona/glv103 PMC4861254

[30] M. Wingood , N. M. Gell , D. E. Rosenberg , G. J. Stoddard , and E. D. Bouldin , “Associations of Cognitively Active Versus Passive Sedentary Behaviors and Cognition in Older Adults,” Journal of Physical Activity & Health 21, no. 9 (2024): 928–938. 39084614 10.1123/jpah.2024-0003 PMC11545599

[31] W. Zhou , K. E. Webster , P. T. Veliz , and J. L. Larson , “Profiles of Sedentary Behaviors in the Oldest Old: Findings From the National Health and Aging Trends Study,” Aging Clinical and Experimental Research 34, no. 9 (2022): 2071–2079. 35676552 10.1007/s40520-022-02157-1

[32] C. Bertuol , K. S. da Silva , A. M. Gerage , et al., “Tempo excessivo de televisão e fatores associados em adultos e idosos da região Sul do Brasil: mudanças após uma década,” Cadernos de Saúde Coletiva 33 (2025): e33010251.

[33] E. C. Lopes , L. M. Cândido , R. A. Rosa , et al., “Tempo de televisão, obesidade e doenças cardiovasculares em idosos brasileiros: Pesquisas Nacionais de Saúde 2013 e 2019,” Ciência & Saúde Coletiva 28 (2023): 3169–3181. 37971001 10.1590/1413-812320232811.12692022

[34] E. A. Rodrigues , A. J. Djiberou Mahamadou , and S. Moreno , “The Impact of Lifestyle Factors on Trajectories of Cognitive Subtypes in the Older Adult Population,” Scientific Reports 15, no. 1 (2025): 31744. 40877588 10.1038/s41598-025-91171-0 PMC12394718

[35] C. Xu , Z. Cao , Z. Lu , Y. Hou , Y. Wang , and X. Zhang , “Associations Between Recreational Screen Time and Brain Health in Middle‐Aged and Older Adults: A Large Prospective Cohort Study,” Journal of the American Medical Directors Association 25, no. 8 (2024): 104990. 38642587 10.1016/j.jamda.2024.03.010

[36] A. Cegolon and A. Jenkins , “Older Adults, Cognitively Stimulating Activities and Change in Cognitive Function,” International Journal of Lifelong Education 41, no. 4–5 (2022): 405–419.

[37] R. J. Dougherty , T. D. Hoang , L. J. Launer , D. R. Jacobs , S. Sidney , and K. Yaffe , “Long‐Term Television Viewing Patterns and Gray Matter Brain Volume in Midlife,” Brain Imaging and Behavior 16, no. 2 (2022): 637–644. 34487279 10.1007/s11682-021-00534-4 PMC8898315

[38] A. A. Mercadante and P. Tadi , “Neuroanatomy, Gray Matter,” in StatPearls (StatPearls Publishing, 2025), http://www.ncbi.nlm.nih.gov/books/NBK553239/. 31990494

[39] H. Takeuchi and R. Kawashima , “Effects of Television Viewing on Brain Structures and Risk of Dementia in the Elderly: Longitudinal Analyses,” Frontiers in Neuroscience 17 (2023): 984919. 36968501 10.3389/fnins.2023.984919 PMC10030518

[40] M. Hallgren , D. W. Dunstan , and N. Owen , “Passive Versus Mentally Active Sedentary Behaviors and Depression,” Exercise and Sport Sciences Reviews 48, no. 1 (2020): 20–27. 31663866 10.1249/JES.0000000000000211

[41] B. L. Hudgins , D. J. Hevel , and J. P. Maher , “Screen‐Based and Non‐Screen‐Based Sedentary Behaviors Are Differentially Associated With Affective States in Older Adults,” Psychology of Sport and Exercise 67 (2023): 102433. 37665886 10.1016/j.psychsport.2023.102433

[42] A. O. Werneck , N. Owen , R. H. O. Araujo , D. R. Silva , and M. Hallgren , “Mentally‐Passive Sedentary Behavior and Incident Depression: Mediation by Inflammatory Markers,” Journal of Affective Disorders 339 (2023): 847–853. 37467803 10.1016/j.jad.2023.07.053

[43] S. Kurita , T. Doi , K. Tsutsumimoto , et al., “Cognitive Activity in a Sitting Position Is Protectively Associated With Cognitive Impairment Among Older Adults,” Geriatrics & Gerontology International 19, no. 2 (2019): 98–102. 30276939 10.1111/ggi.13532 PMC7379533

[44] R. Wang , M. Liu , X. Cheng , Y. Wu , A. Hildebrandt , and C. Zhou , “Segregation, Integration, and Balance of Large‐Scale Resting Brain Networks Configure Different Cognitive Abilities,” National Academy of Sciences of the United States of America 118, no. 23 (2021): e2022288118. 10.1073/pnas.2022288118 PMC8201916 34074762

[45] L. Y. Lee , M. P. Healy , N. L. Fischer , et al., “Cognitive Flexibility Training for Impact in Real‐World Settings,” Current Opinion in Behavioral Sciences 59 (2024): 101413.

[46] C. Matrisciano , R. Minino , A. M. Mariani , and C. D'Anna , “The Effect of Physical Activity on Executive Functions in the Elderly Population: A Systematic Review of Randomized Controlled Trials,” Brain Sciences 15, no. 7 (2025): 703. 40722295 10.3390/brainsci15070703 PMC12293948

[47] I. Gómez‐Soria , I. Iguacel , A. Aguilar‐Latorre , et al., “Cognitive Stimulation and Cognitive Results in Older Adults: A Systematic Review and Meta‐Analysis,” Archives of Gerontology and Geriatrics 104 (2023): 104807. 36116285 10.1016/j.archger.2022.104807

[48] H. Dejakaisaya , W. Mahikul , N. Na‐Ek , and C. Hirunpattarasilp , “Television Watching and Cognitive Outcomes in Adults and Older Adults: A Systematic Review and Dose‐Response Meta‐Analysis of Observational Studies,” PLoS One 20, no. 9 (2025): e0323863, 10.1371/journal.pone.0323863. 40938929 PMC12431243

[49] D. Fancourt and A. Steptoe , “Television Viewing and Cognitive Decline in Older Age: Findings From the English Longitudinal Study of Ageing,” Scientific Reports 9, no. 1 (2019): 2851, 10.1038/s41598-019-39354-4. 30820029 PMC6395805

[50] M. E. Echevarría‐Guanilo , N. Gonçalves , and P. J. Romanoski , “Psychometric Properties of Measurement Instruments: Conceptual Basis and Evaluation Methods—Part II,” Texto & Contexto—Enfermagem 28 (2019): e20170311.

[51] C. L. Kimberlin and A. G. Winterstein , “Validity and Reliability of Measurement Instruments Used in Research,” American Journal of Health‐System Pharmacy 65, no. 23 (2008): 2276–2284. 19020196 10.2146/ajhp070364

[52] E. N. Baranski , G. Gardiner , E. Guillaume , et al., “Comparisons of Daily Behavior Across 21 Countries,” Social Psychological and Personality Science 8, no. 3 (2017): 252–266.

[53] J. Li , D. Y. T. Fong , K. Y. W. Lok , et al., “Country‐Specific Key Lifestyle Factors and Health Outcomes for Resource Allocation in the General Population: A Network Analysis Across 29 Countries,” Journal of Global Health 15 (2025): 04011. 39791329 10.7189/jogh.15.04011 PMC11719263

